# Maybe She’ll Make Some Friends?: Family Members Cultivating Intimacy in Assisted Living

**DOI:** 10.1093/geroni/igaa057.1573

**Published:** 2020-12-16

**Authors:** Andrea Fitzroy, Candace Kemp, Elisabeth Burgess

**Affiliations:** Georgia State University, Atlanta, Georgia, United States

## Abstract

Intimacy continues to be important in later life, including for older adults in long-term care settings such as assisted living (AL). Our past work shows that intimacy is a multi-dimensional process and can involve a variety of partners. Drawing on data from the qualitative longitudinal “Convoys of Care” study (R01AG044368), we extend this research to examine the role family members play in cultivating intimacy and close relationships of AL residents. Using a grounded theory approach, we analyzed 2,224 hours of participant observation, and formal interviews with 28 assisted living residents (aged 58-96) and their formal and informal care partners (n=114) from four diverse AL communities. Findings show that family members can play integral roles in residents’ experiences with intimacy, directly as relationship partners, and by facilitating or impeding residents’ contacts with others. Family members cultivated residents’ intimacy opportunities and experiences by direct engagement, resident advocacy, to non-involvement and disengagement. Family members’ roles in cultivating intimacy fluctuated over time, increasing at times of health concerns and family change. Perceptive family members considered older adults’ intimacy preferences when cultivating their intimate relationships. Family members concerned for the safety of their loved one sometimes acted as “gatekeepers” to intimacy by interfering in intimate relationships. We conclude with a discussion of implications for policy and practice aimed at improving the intimacy process and opportunities for older adults receiving long-term care.

